# Quality control and considering systematic MIC shifts are key when evaluating the role of *mmpR5* (*Rv0678*) frameshifts in bedaquiline resistance

**DOI:** 10.1128/aac.00266-25

**Published:** 2025-08-14

**Authors:** Thomas Schön, Paolo Miotto, Varvara K. Kozyreva, Matthew D. Sylvester, Daniela M. Cirillo, Claudio U. Köser

**Affiliations:** 1 Department of Biomedical and Clinical Sciences, Division of Infection and Inflammation, Linköping University 568027, Linköping, Sweden; 2 Department of Infectious Diseases, Kalmar County Hospital, Linköping University 4566, Kalmar, Sweden; 3 Department of Infectious Diseases, Linköping University Hospital 4566, Linköping, Sweden; 4 Emerging Bacterial Pathogens Unit, IRCCS San Raffaele Scientific Institute, Milan, Italy; 5 Microbial Diseases Laboratory Branch, California Department of Public Health, Richmond, California, USA; 6 Department of Genetics, University of Cambridge 98529, Cambridge, United Kingdom; St George's, University of London, London, United Kingdom

**Keywords:** *Mycobacterium tuberculosis*, genotypic antimicrobial susceptibility testing, bedaquiline

## LETTER

The accurate interpretation of genotypic antimicrobial susceptibility testing (gAST) results for bedaquiline for *Mycobacterium tuberculosis* is crucial as it is a key drug in most 6- to 9-month all-oral regimens for rifampicin-resistant tuberculosis (1, 2). Unfortunately, a large spectrum of resistance mutations is possible in *mmpR5* (*Rv0678*), the most important bedaquiline resistance mechanism that also confers cross-resistance with clofazimine. To improve the sensitivity of gAST, WHO endorsed an additional grading rule, whereby any loss-of-function in *mmpR5* (i.e., full-gene deletion, frameshifts, mutations that abolish the start codon, and premature stop codons) should be interpreted as an interim resistance mutation, taking epistasis caused by loss-of-function mutations in *mmpL5* into consideration where possible (3, 4).

WHO and others acknowledged that exceptions to these additional grading rules may exist and should be studied because they may overcall resistance (3, 5). Snobre et al. recently proposed that some *mmpR5* frameshifts subjected to the additional grading rule might retain susceptibility to bedaquiline by producing functional MmpR5 through alternative reading frames (1). However, when reviewing the Becton Dickinson (BD) BACTEC Mycobacteria Growth Indicator Tube 960 (MGIT) MIC data used to support their hypothesis, we identified several factors that should be considered.

49% (33/68) of the frameshift MGIT MICs analyzed were from Peretokina et al., which includes data from Zimenkov et al. from the same laboratory, whereas the remaining results were from three separate laboratories (1, 6–10). Only one of the 33 frameshift MICs (3%, 95% confidence interval [CI] 0–16) from Peretokina et al. was above the current interim critical concentration (CC) of 1 µg/mL, whereas 31 of the remaining 35 frameshift MICs (89%, 95% CI 73–97) from the remaining studies were in the resistant range (1). This suggested that the MGIT MICs in Peretokina et al. were systematically low, which was acknowledged by the authors and is in line with the following observations (8).

First, although no formal quality control (QC) range for the H37Rv control strain currently exists for bedaquiline, the MGIT MICs from Peretokina et al. appeared to be shifted towards lower values compared with the data considered by WHO to set the current CC for MGIT (8, 11).

Second, the 148 MGIT MICs of isolates without *mmpR5* or *atpE* mutations prior to bedaquiline treatment from Peretokina et al. were between ≤0.03 and 0.25 µg/mL with a mode at 0.125 µg/mL (Table S1), which was lower than the modes of 0.25 or 0.5 µg/mL observed in the MIC distributions used by WHO (8, 11). The equivalent mode for 7H11 was ≤0.03 µg/mL (Table S1) and thus again lower than 0.06 µg/mL in the WHO distributions (8, 11).

Third, 12 of the 15 isolates with MGIT MICs in the resistant range either had or were later found to have an *atpE* mutation (Table S2) (8). For 7H11, the same applied to seven of the eight MICs in the resistant range (Table S2). Given that *atpE* mutations typically confer larger MIC increases than *mmpR5*, this indicates systematic shifts towards lower MICs for both media (12).

Fourth, 17 of 33 isolates with *mmpR5* frameshifts MICs that Snobre et al. included from Peretokina et al. arose during bedaquiline treatment of seven patients who had genotypically wild-type isolates at baseline. Notably, the detection of the frameshift usually correlated with an MIC increase in MGIT as well as 7H11 (Table S3) (8). This is evidence of positive selection and means that epistasis linked to *mmpL5* can be excluded as a confounder (3, 13).

Systematic MIC shifts have been described for other anti-TB agents in the past and, depending on their magnitude and direction, are difficult to detect if only the CC is tested or MIC distributions are not scrutinized (11, 14, 15). Instead, the inclusion of a QC strain that yields an on-scale MIC is key to detect shifts towards lower or higher MICs (16). For example, a BD BACTEC PZA reagent quality issue was detected early by the California Department of Public Health because of abnormally high MICs for H37Rv with various lots of reagents, which coincided with an increased frequency of likely false-resistant phenotypic results for clinical strains without gAST support. BD’s user QC protocol, which recommends testing only the CC, initially did not reveal any problems (17). Later, false resistance for PZA was noted globally, ultimately resulting in a recall of affected reagent lots by BD (18). This underlines that all manufacturers of pAST devices for anti-TB agents should establish QC ranges/targets that should be published for internal as well as external QC. Indeed, EUCAST calls for setting QC ranges/targets for the EUCAST reference MIC method as well as any other method that is calibrated against the reference method (19).

In summary, the *in vitro* evidence for susceptibility supporting the hypothesis by Snobre et al. was largely from Peretokina et al., which should have been excluded from the analysis because of the likely systematic MIC shift that resulted in false-susceptible results using the current CC (1, 8). Given the clinical implications of their hypothesis, additional data sets with appropriate QC results should be interrogated to study this important question.

## SUPPLEMENTAL MATERIAL

Supplemental tables aac.00266-25-s0001.xlsx Tables S1 to S3.

ASM does not own the copyrights to Supplemental Material that may be linked to, or accessed through, an article. The authors have granted ASM a non-exclusive, world-wide license to publish the Supplemental Material files. Please contact the corresponding author directly for reuse.

## References

[1] Snobre J, Meehan CJ, Mulders W, Rigouts L, Buyl R, de Jong BC, Van Rie A, Tzfadia O. 2024. Frameshift mutations in the mmpR5 gene can have a bedaquiline-susceptible phenotype by retaining a protein structure and function similar to wild-type Mycobacterium tuberculosis Antimicrob Agents Chemother 68:e0085424. doi:10.1128/aac.00854-24 39445816 PMC11619236

[2] World Health Organization. 2025. WHO consolidated guidelines on tuberculosis. Module 4: treatment and care. Available from: https://iris.who.int/handle/10665/380799 42866503

[3] World Health Organization. 2023. Catalogue of mutations in Mycobacterium tuberculosis complex and their association with drug resistance, 2nd ed. Available from: https://iris.who.int/handle/10665/374061

[4] Miotto P, Cirillo DM, Schön T, Köser CU. 2024. The exceptions that prove the rule–a historical view of bedaquiline susceptibility. Genome Med 16:39. doi:10.1186/s13073-024-01311-w 38481348 PMC10935885

[5] Köser CU, Miotto P, Ismail N, Anthony RM, Utpatel C, Merker M, Niemann S, Tahseen S, Rigouts L, Rodrigues C, Omar SV, Farhat MR, Antonenka U, Hoffmann H, Cirillo DM, Schön T. 2024. A composite reference standard is needed for bedaquiline antimicrobial susceptibility testing for Mycobacterium tuberculosis complex. Eur Respir J 64:2400391. doi:10.1183/13993003.00391-2024 38991722 PMC11237371

[6] Zimenkov DV, Nosova EYu, Kulagina EV, Antonova OV, Arslanbaeva LR, Isakova AI, Krylova LYu, Peretokina IV, Makarova MV, Safonova SG, Borisov SE, Gryadunov DA. 2017. Examination of bedaquiline- and linezolid-resistant Mycobacterium tuberculosis isolates from the Moscow region. J Antimicrob Chemother 72:1901–1906. doi:10.1093/jac/dkx094 28387862

[7] Ghodousi A, Rizvi AH, Baloch AQ, Ghafoor A, Khanzada FM, Qadir M, Borroni E, Trovato A, Tahseen S, Cirillo DM. 2019. Acquisition of cross-resistance to bedaquiline and clofazimine following treatment for tuberculosis in Pakistan. Antimicrob Agents Chemother 63:e00915-19. doi:10.1128/AAC.00915-19 31262765 PMC6709449

[8] Peretokina IV, Krylova LY, Antonova OV, Kholina MS, Kulagina EV, Nosova EY, Safonova SG, Borisov SE, Zimenkov DV. 2020. Reduced susceptibility and resistance to bedaquiline in clinical M. tuberculosis isolates. J Infect 80:527–535. doi:10.1016/j.jinf.2020.01.007 31981638

[9] Conradie F, Diacon AH, Ngubane N, Howell P, Everitt D, Crook AM, Mendel CM, Egizi E, Moreira J, Timm J, McHugh TD, Wills GH, Bateson A, Hunt R, Van Niekerk C, Li M, Olugbosi M, Spigelman M, Nix-TB Trial Team. 2020. Treatment of highly drug-resistant pulmonary tuberculosis. N Engl J Med 382:893–902. doi:10.1056/NEJMoa1901814 32130813 PMC6955640

[10] Brown TS, Tang L, Omar SV, Joseph L, Meintjes G, Maartens G, Wasserman S, Shah NS, Farhat MR, Gandhi NR, Ismail N, Brust JCM, Mathema B. 2024. Genotype-phenotype characterization of serial Mycobacterium tuberculosis isolates in bedaquiline-resistant tuberculosis. Clin Infect Dis 78:269–276. doi:10.1093/cid/ciad596 37874928 PMC11494438

[11] World Health Organization. 2018. Technical report on critical concentrations for drug susceptibility testing of medicines used in the treatment of drug-resistant tuberculosis. Available from: https://iris.who.int/handle/10665/260470

[12] Kadura S, King N, Nakhoul M, Zhu H, Theron G, Köser CU, Farhat M. 2020. Systematic review of mutations associated with resistance to the new and repurposed Mycobacterium tuberculosis drugs bedaquiline, clofazimine, linezolid, delamanid and pretomanid. J Antimicrob Chemother 75:2031–2043. doi:10.1093/jac/dkaa136 32361756 PMC7825472

[13] Vargas R, Freschi L, Spitaleri A, Tahseen S, Barilar I, Niemann S, Miotto P, Cirillo DM, Köser CU, Farhat MR. 2021. Role of epistasis in amikacin, kanamycin, bedaquiline, and clofazimine resistance in Mycobacterium tuberculosis Complex. Antimicrob Agents Chemother 65:e01164–21. doi:10.1128/AAC.01164-21 34460306 PMC8522733

[14] World Health Organization. 2021. Technical report on critical concentrations for drug susceptibility testing of isoniazid and the rifamycins (rifampicin, rifabutin and rifapentine). Available from: https://iris.who.int/handle/10665/339275

[15] Kahlmeter G, Turnidge J. 2023. Wild-type distributions of minimum inhibitory concentrations and epidemiological cut-off values–laboratory and clinical utility. Clin Microbiol Rev 36:e0010022. doi:10.1128/cmr.00100-22 38038445 PMC10732016

[16] Schön T, Matuschek E, Mohamed S, Utukuri M, Heysell S, Alffenaar J-W, Shin S, Martinez E, Sintchenko V, Maurer FP, Keller PM, Kahlmeter G, Köser CU. 2019. Standards for MIC testing that apply to the majority of bacterial pathogens should also be enforced for Mycobacterium tuberculosis complex. Clin Microbiol Infect 25:403–405. doi:10.1016/j.cmi.2019.01.019 30771527 PMC7903878

[17] BD. 2019. BACTEC^TM^ MGIT^TM^ 960 PZA Kit for the antimycobacterial susceptibility testing of Mycobacterium tuberculosis. Available from: https://eifu.bd.com/hcp/ids/US/general?keycode=245128

[18] BD. 2024. Urgent: medical device correction. type of action: product removal – discarded by customer BD BACTEC^TM^ MGIT^TM^ 960 PZA Kit. Available from: https://bdx.my.salesforce.com/sfc/p/?ACSTrackingID=DM134407&ACSTrackingLabel=Lab%20Alert%3A%20BD%20Life%20Sciences%20Issues%20Notice%20to%20Immediately%20Discard%20Affected%20BD%20BACTEC%20MGIT%20960%20PZA%20Kits&deliveryName=DM134407#30000000Y0Yr/a/8a000001prR0/vij3mip7nb40S80cG4xGLyKAO.1tQSBS0LhkUbwpLdA

[19] Schön Thomas, Köser CU, Werngren J, Viveiros M, Georghiou S, Kahlmeter G, Giske C, Maurer F, Lina G, Turnidge J, van Ingen J, Jankovic M, Goletti D, Cirillo DM, Santin M, Cambau E. 2020. What is the role of the EUCAST reference method for MIC testing of the Mycobacterium tuberculosis complex? Clin Microbiol Infect 26:1453–1455. doi:10.1016/j.cmi.2020.07.037 32768492

